# L-serine metabolic regulation and host respiratory homeostasis

**DOI:** 10.3389/fcimb.2025.1518659

**Published:** 2025-02-26

**Authors:** Pan Li, Xiaoyan Wu, Yanlan Huang, Ruijing Qin, Pan Xiong, Yangyang Qiu

**Affiliations:** ^1^ Department of Environment and Safety Engineering, Taiyuan Institute of Technology, Taiyuan, China; ^2^ National Engineering Research Center of Immunological Products, Department of Microbiology and Biochemical Pharmacy, College of Pharmacy, Third Military Medical University, Chongqing, China; ^3^ College of Veterinary Medicine, Southwest University, Chongqing, China; ^4^ College of Agronomy and Biotechnology, Southwest University, Chongqing, China

**Keywords:** serine, inflammation, infection, respiratory diseases, PHGDH

## Abstract

L**-**Serine, a non-essential amino acid (NEAA), can be obtained through diet or *in situ* synthesis. Functionally, L-serine not only serves as the precursor of other amino acids, lipids, and nucleotides, but also participates in the folate/methionine cycle. An increasing number of studies have demonstrated that L-serine is widely used in the adjuvant therapy of many diseases (e.g., inflammation, infections, fibrosis, and tumors). Here, we summarize the synthesis and metabolism of serine followed by its functions in health and disease. Moreover, we delineate the potential mechanisms whereby L-serine is involved in the occurrence and progression of respiratory diseases. This review aims to summarize the research progress of serine in diseases, propose the problems that need to be solved in the future, and provide guidance for subsequent research and development.

## 1 Introduction

Amino acids are one of the many biologically active macromolecules that build biological organisms and are the basic materials for cell structure and tissue repair (Rusciano et al., 2016). As one of the basic structural substances constituting the immune system, amino acids contribute to the development of immune organs, the proliferation and differentiation of immune cells, and the secretion of cytokines, all of which regulate the immune response (Kelly and Pearce, 2020). Insufficient intake of amino acids results in immune organ atrophy and impaired immune cell function. Rational supplementation of amino acids has a positive effect on the regulation of the immune function of the body (Lieu et al., 2020).

Serine, including D-serine and L-serine, is known as non-essential amino acid (NEAA), which is synthesized in the body from glycine and 3-phosphoglyceric acid (Lee et al., 2018), and L-serine can be converted to D-serine by serine racemase (SR) (Wolosker, 2018). L-serine has numerous functions, including the production of small molecules involved in cellular metabolism, for example: as the precursors for the synthesis of amino acids (e.g., glycine, cysteine, and taurine), nucleotides (e.g., purine and pyrimidine nucleotides), neurotransmitters, and phospholipids (e.g., sphingolipids and phosphatidylserine), as well as a methyl donor for protein and DNA synthesis (Murtas et al., 2020). Similarly, serine plays a critical role in the regulation of the immune system and prevention of diseases. Yu reported that serine metabolism can inhibit ATP6V0D2-mediated YAP lysosomal degradation, thereby regulating antiviral innate immunity (Shen et al., 2021). Furthermore, L-serine has been found to have beneficial effects in the treatment of neurological conditions such as depression, schizophrenia, chronic fatigue syndrome, and intellectual disability (Wolosker and Radzishevsky, 2013; Zhang et al., 2018b), as well as alcoholic and non-alcoholic fatty liver disease (Sim et al., 2015). It is worth noting that L-serine supplementation is not necessarily the gold standard for treating diseases, but sometimes restricting L-serine supplementation is beneficial for treating diseases (Maddocks et al., 2017). Therefore, maintaining appropriate serine intake is essential for maintaining normal protein synthesis, metabolism, and neurological function. In addition to these physiological functions, L-serine is also widely used as a moisturizer and antioxidant in medications, sports drinks, and cosmetics (Zhang et al., 2018b).

Here, we summarize the synthesis and metabolism of serine followed by the functions of serine in health and disease. Meanwhile, we delineate the potential mechanisms whereby L-serine influences the occurrence and progression of various diseases. We expect that this review will provide guidance for subsequent research and development in the treatment of inflammatory and respiratory diseases.

## 2 L-serine and metabolic regulation

### 2.1 L-serine forms a complex metabolic network

**Figure 1:**
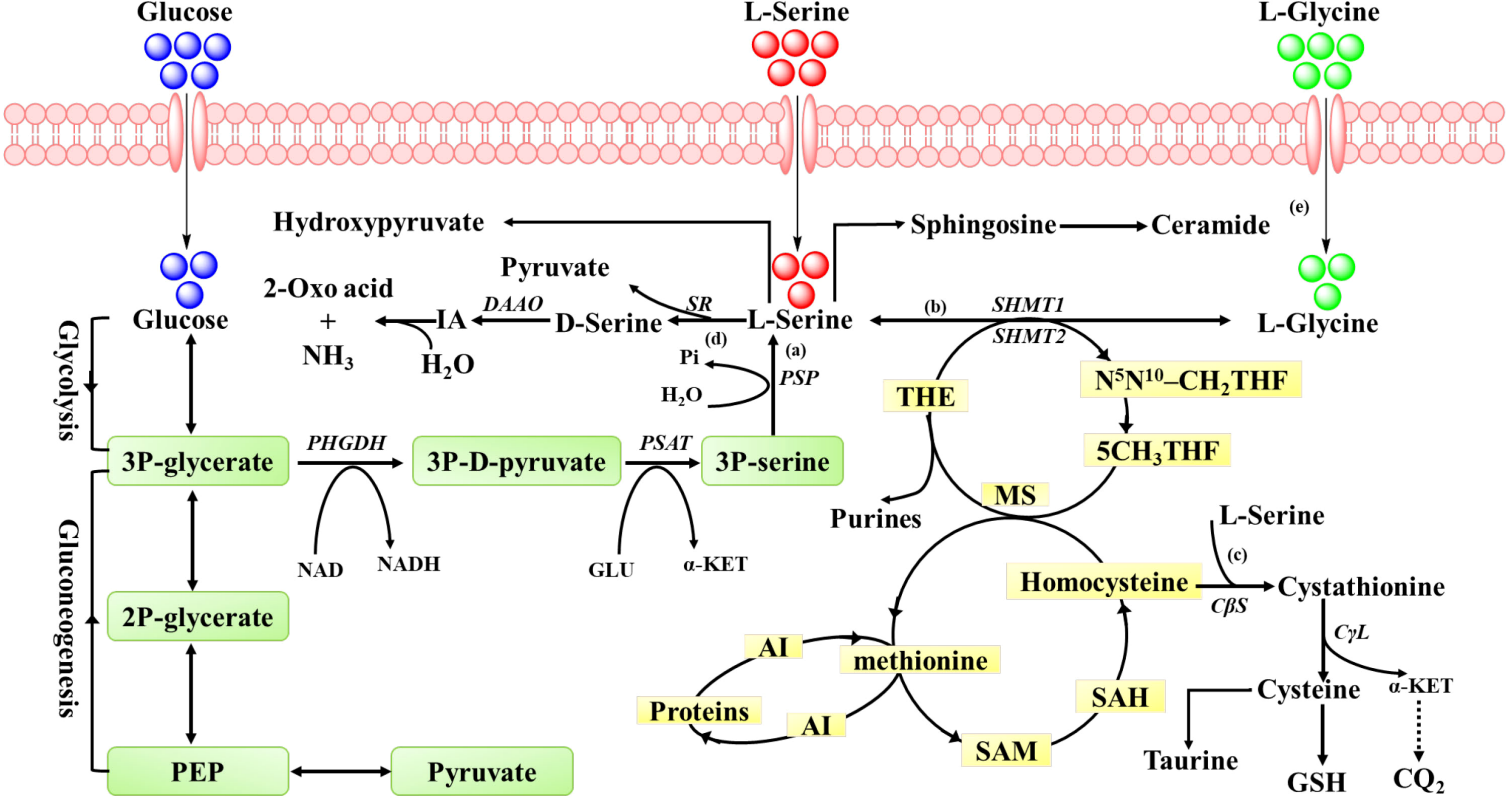
Pathways of serine synthesis and metabolism. **(a)** The intermediates in the pathways shown in green are involved in the synthesis of L-serine, either from glucose via glycolysis or from the gluconeogenic intermediate 3P-glycerate. L-serine synthesis involves three main steps. The first step is that PHGDH catalyzes 3P-glycerate to 3P-D-pyruvate. The next step is that the PSAT converts 3P-D-pyruvate into 3P-serine. The last step is serine synthesis through hydrolysis of 3P-serine catalyzed by PSP. **(b)** L-serine can also be obtained from glycine via a process catalyzed by SHMT_2_. **(c)** L-serine metabolism involves two main metabolic pathways. One is the synthesis of glycine, cystine, taurine and GSH through the carbon cycle. **(d)** In the second metabolic pathway of L-serine, SR catalyzes the conversion of L-serine to D-serine, which is oxidized by D-amino acid oxidase to generate amino acid, which is then non-enzymatic hydrolyzed to the corresponding 2-oxic acid and ammonia. **(e)** L-serine is metabolized to pyruvate through a non-phosphorylated pathway. Serine is also involved in the formation of phospholipids. 3P-glycerate, 3-phosphoglycerate; PHGDH, 3-phosphoglycerate dehydrogenase; 3P-D-pyruvate, 3-phosphohydroxypyruvate; PSAT, 3-phosphoserine aminotransferase; GLU, glutamate; α-KET, α-ketoglutarate; 2P-glycerate, 2-phosphoglycerate; 3P-serine, 3-phosphoserine; Pi, inorganic phosphate7; PSP, 3-phosphoserine phosphatase; FA, Fatty acids; AI, Amino acids; DAAO, D-amino-acid oxidase; PEP, phosphoenolpyruvate; MS, methionine synthase; SAM, S-adenosylmethionine; SAH, S-adenosylhomocysteine; CβS; cystathionine β-synthase; CγL, cystathionine γ-lyase; N^5^N^10^–CH_2_THF, 5,10-methylene-tetrahydrofolate; 5CH_2_THF, 5-methylene-tetrahydrofolate; GSH, glutathione.

As a glycogenic amino acid, L-serine has various sources, such as an external diet, intracellular synthesis (from the glycolytic intermediate 3-phosphoglycerate and glycine), and cleavage of proteins and phospholipids *in vivo* (Kishor et al., 2020). Owing to the existence of the blood-brain barrier, L-serine absorbed from the diet cannot meet the needs of the brain and thus must be synthesized *in situ* (Yoshida et al., 2004). During *de novo* synthesis, L-serine is converted from 3-phosphoglycerate (3-PG), an intermediate of glycolysis, through a three-step enzymatic reaction, in which 3-phosphoglycerate dehydrogenase (PHGDH) is the first rate-limiting enzyme (**Figure 1a**) (Mattaini et al., 2016; Shen et al., 2021). L-serine, on the other hand, is reversibly catalyzed to glycine and CH2-THF by SHMT1 or SHMT2 in the cytosol or mitochondria, respectively, and participates in one-carbon metabolism and the S-adenosine methionine (SAM) metabolic network through the transfer of one-carbon units (**Figure 1b**) (Locasale, 2013; Mattaini et al., 2016).

Therefore, L-serine is a precursor molecule for the synthesis of many substances, and the metabolism of L-serine includes the synthesis of amino acids, phospholipids, and proteins (Holeček, 2022). First, serine, as the main substrate, plays an important role in protein synthesis (Reeds, 2000). In addition, L-serine mainly synthesizes glycine and cysteine via transsulfuration to participate in the formation of glutathione (GSH), which plays significant roles in redox reactions (**Figure 1c**) (Zhang et al., 2018a; Zhang et al., 2019). Moreover, L-serine is involved in the synthesis of phospholipids and glycolipids, which are important components of cell membranes and highly direct cell differentiation, proliferation, and apoptosis (Murtas et al., 2020). In addition, L-serine is a major carbon unit donor of the folate cycle that produces NADPH, NADH and ATP in the one-carbon metabolic reaction, as well as the main carbon unit donor in the methionine cycle to synthesize adenosine, guanosine, and thymine (Ducker and Rabinowitz, 2017; Kim and Park, 2018), and a methyl donor for protein and DNA synthesis (**Figure 1d**) (Murtas et al., 2020). Collectively, the synthesis and metabolism of serine are summarized in **Figure 1**.

### 2.2 Transformation from L-serine to its isomer D-serine

In 1992, Hashimoto et al. first reported the presence of large amounts of free D-serine, the optical isomer of L-serine, in mammalian brain tissue (Hashimoto et al., 1992). Later, Schell and Williams et al. found that the distribution of D-serine varied regionally, i.e. it was clearly located in the astrocytoid cell subpopulation (Schell et al., 1995), which also implied that D-serine may be involved in a biosynthetic pathway. Wolosker and colleagues purified serine racemase, which can directly racemate L-serine to form D-serine, and found that multiple properties of SR were similar to those of bacterial racemase, suggesting that the D-amino acid biosynthesis pathway is conserved from bacterial to mammalian brains (Wolosker et al., 1999).

The metabolism of D-serine is catalyzed mainly by D-amino acid oxidase (DAAO) bonding flavin adenine dinucleotide (FAD) (**Figure 1e**) (Pollegioni and Sacchi, 2010). A comparative study of wild-type (WT) and SR-knockout (SR-KO) mice revealed that the SR is involved primarily in D-serine production in the forebrain of mice, and that there might be other D-serine production pathways may be involved in the brain and peripheral organs (Horio et al., 2011). Due to differences in tissue culture and immunocytochemical techniques, there is still no clear conclusion on whether D-serine is distributed in astroglia or neurons, and Coyle et al. have already made a clear discussion on this issue (Coyle et al., 2020); we will not go into the details here.

A study demonstrated that an important rate-limiting factor in maintaining D-serine balance in the brain is L-serine synthesis via the phosphorylation pathway, which is modeled in mice with astrocyte conditions lacking Phgdh. Moreover, D-serine deficiency restricts the function of NMDA receptors (NMDARs) (Yang et al., 2010). D-serine is an endogenous co-agonist of NMDARs and is required to regulate synaptic plasticity and excitatory transmission in the central nervous system (CNS) (Mothet et al., 2000; Panatier et al., 2006). In addition, some studies have proposed the concept of a “serine shuttle” in astrocyte-neuron metabolism, in which D-serine indirectly regulates NMDAR (Bonvento and Bolaños, 2021; Wolosker and Radzishevsky, 2013). Therefore, some studies have shown that the level of D-serine in the host may be related to neurological diseases, such as Alzheimer’s disease, cognitive dysfunction, schizophrenia, depression, and addiction (Holeček, 2022).

However, D-serine has been reported to be nephrotoxic and neurotoxic. For example, astrocytes in primary cultures express SR, synthesize D-serine and acquire A1 (an excitotoxic moiety released from inflammation) reactive astrocyte features (Coyle et al., 2020). Hippocampal synaptic damage in mouse caused by controlled cortical impact resulted in the conversion of D-serine release from neurons to astrocytes, further exacerbating synaptic damage and dysfunction (Perez et al., 2017). On the other hand, D-serine mainly causes dose-related (≥500 mg/kg) nephrotoxicity in rats, manifested as reversible acute tubular necrosis (Okada et al., 2017). However, compared with humans, the ratio of the oral dose to the serum concentration seems to be higher in rats than in humans, making it difficult to directly apply relevant studies to humans (Meftah et al., 2021). In conclusion, L-serine, as a precursor of D-serine synthesis, is involved in the regulation of D-serine level, so the transformation from L-serine to D-serine is of great significance to human health.

### 2.3 L-serine exerts biological functions by regulating metabolism

As previously mentioned, L-serine participates in complex metabolic networks in the body, so it has the potential to regulate cellular processes such as cell proliferation and immune cell activation by regulating the metabolic microenvironment and nutrient availability. For example, L-serine provides glycine and one carbon unit to effector T (Teff) cells and promotes their proliferation independently of glycolysis (Ma et al., 2017). In addition, L-serine supports cell proliferation and maintains mitochondrial function through ceramide metabolism (Gao et al., 2018). On the other hand, L-serine is catalyzed by serine palmitoyl transferase (SPT) to synthesize sphinganine and eventually generate ceramide, which plays a role in neuronal genesis and survival (Muthusamy et al., 2020). When alanine is the substrate, SPT catalyzes the production of toxic 1-deoxysphinglipids (doxSLs), which may induce neuropathy (Muthusamy et al., 2020).

Interestingly, L-serine regulates growth hormone and corticosterone concentrations, which may be related to its metabolic rhythm (Wu et al., 2019). The concentration of L-serine in cerebrospinal fluid (CSF) decreases with age from a mean of 59 μmol/L at the age of 1 week to a mean of 31 μmol/L at the age of 10 years, suggesting that the demand for L-serine varies with age (van der Crabben et al., 2013). Moreover, L-serine can enhance the circadian phase resetting of mice and humans induced by light, and supplementation with L-serine is helpful for human sleep (Ito et al., 2014). In mammals, the suprachiasmatic nucleus (SCN) of the hypothalamus is the pacemaker that regulates circadian rhythms (Yan et al., 2020). L-serine alters the long-term expression pattern of the SCN clock gene through GABA_A_ receptors and enhances light-induced phase resetting in mice and humans, so the combination of L-serine and light therapy may help treat circadian rhythm disturbances (Yasuo et al., 2017).

## 3 L-serine and host health homeostasis

### 3.1 The role of L-serine in stress and inflammatory responses

**Figure 2:**
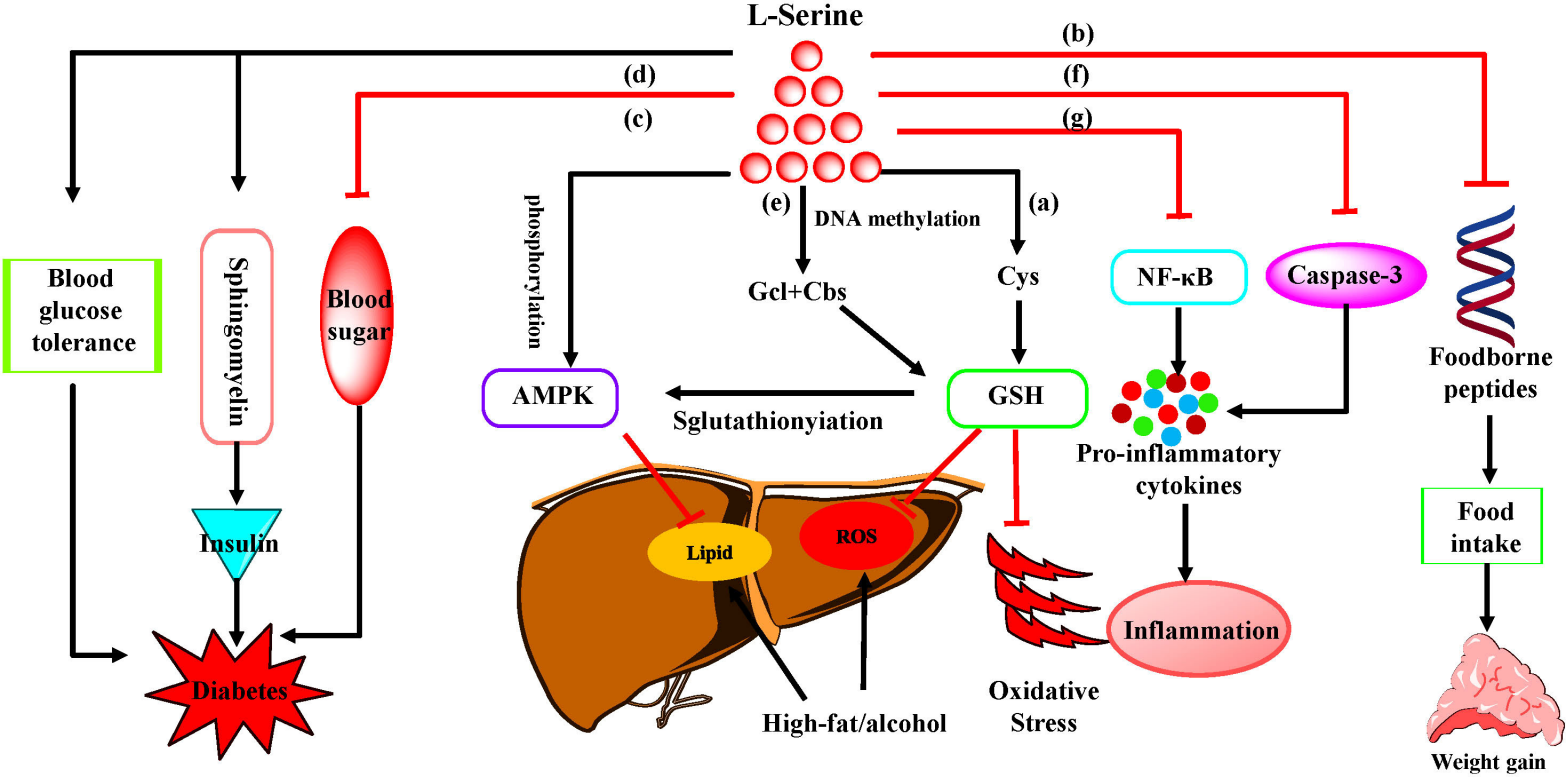
The role and mechanism of L-serine in host homeostasis. **(a)** L-serine is used to prevent and treat fatty liver diseases by increasing homocysteine metabolism. **(b)** L-serine significantly reduces ingestion and weight gain by reducing the expression of foodborne peptides. **(c)** L-serine addition reduces blood sugar and improves blood glucose tolerance, thereby reducing the incidence of type 1 diabetes in Non-obese diabetes (NOD) mice. **(d)** L-serine improves blood glucose tolerance, thus reducing the incidence of diabetes by regulating the composition of sphingomyelin, which regulates insulin folding, proliferation and apoptosis. **(e)** L-serine inhibits oxidative stress by increasing GSH and activating the AMPK pathway. **(f)** L-serine reduces macrophage- and neutrophil-mediated inflammatory responses by inhibiting inflammasomes. **(g)** L-serine significantly reduces the concentrations of inflammatory cytokines (e.g. TNF-α, IL-1β, IL-6 and IL-8) via the NF-κB signaling pathway.

As a potential anti-stress factor, L-serine not only decreases the production of ROS induced by cisplatin (Monroe et al., 2021) but also attenuates the stress response of neonatal chicks (He et al., 2021); accordingly, researchers suspect that L-serine may be an anti-stress factor. Therefore, studies have investigated the effects of L-serine on the behavior of animals exposed to chronic stress, and the results suggest that the oral administration of L-serine reduces the locomotor activity of socially isolated rats (Shigemi et al., 2010). In addition, L-serine can attenuate the stress response of neonatal chicks under acute stress conditions (Kurauchi et al., 2009). Therefore, L-serine may have application value as an anti-stress factor. With respect to the underlying mechanism, a series of studies have shown that L-serine administration attenuates oxidative stress by increasing catalase (CAT), GSH peroxidase (GSH-Px), superoxide dismutase (SOD) and diamine oxidase (DAO) levels while decreasing apoptosis and malondialdehyde (MDA) levels (He et al., 2023; Zhou et al., 2018a; Zhou et al., 2018c). This is achieved mainly by contributing to the methionine cycle (Zhou et al., 2017) and the SIRT1 pathway (Zhou et al., 2018b), inhibiting hypermethylation of promoter associated with GSH synthesis-related genes, and activating the adenosine 5’-monophosphate (AMP)-activated protein kinase (AMPK) pathway (Zhou et al., 2018a). Therefore, L-serine is a relatively effective small molecule for the treatment of oxidative stress with a low risk of side effects (**Figure 2a**).

Obesity is a disease associated with chronic inflammation, oxidative stress, insulin resistance, unbalanced nutrition and other factors (Gasmi et al., 2021). The addition of L-serine to pregnant mice can change the composition of free amino acids in maternal milk and reduce the weight of offspring (Sim et al., 2015). L-serine significantly reduces ingestion and weight gain by reducing the expression of foodborne peptides in aging mice (Zhou et al., 2018c) (**Figure 2b**). However, maternal dietary serine supplementation could improve the nutritional status of sows and their offspring, which might contribute to the increased body weight of offspring (Zhou et al., 2022a). Thus, more experimental studies are necessary to analyze the specific role of serine in obesity.

Additionally, clinical samples revealed that serum L-serine levels were lower in patients with type 2 diabetes and gestational diabetes than in those without (Holm and Buschard, 2019), and L-serine addition reduced blood sugar and improved blood glucose tolerance, thereby reducing the incidence of type 1 diabetes in Non-obese diabetes (NOD) mice (Rotstein et al., 2010) (**Figure 2c**). Moreover, sphingolipid, which is synthesized from L-serine and palmitoyl-CoA (Rotstein et al., 2010), has important functions in regulating insulin folding, secretion, proliferation and apoptosis (Holm et al., 2018). Therefore, the beneficial effect of L-serine supplementation on diabetes may involve regulating the composition of complex sphingolipids (**Figure 2d**), but a more detailed mechanism needs to be further studied. Collectively, L-serine could exert its effects on obesity and diabetes via various potential mechanisms (**Figures 2b–d**).

Inflammation is a complex process of the immune response (Elinav et al., 2013). In addition to controlling infection and promoting tissue repair, hyperinflammation can also cause tissue damage and disease (Reijnders et al., 2020). Therefore, excessive inflammation needs to be controlled by drugs or other clinical methods to avoid tissue damage (Reijnders et al., 2020). However, medications often have side effects and require the supervision of a doctor. Thus, uncovering the potential role of existing natural nutrients in the treatment of inflammatory diseases could provide new directions for safe drug use.

As mentioned above, L-serine has anti-inflammatory effects and has been proved to have certain therapeutic effects on a variety of inflammation-related diseases, such as fatty liver, obesity, and diabetes (Zhang et al., 2021). For example, drugs containing L-serine can be used to prevent and treat fatty liver diseases according to a patent (Lee and Yin, 2011), and L-serine alone further illustrates the potential of L-serine to treat fatty liver disease by reducing alcohol-induced hepatic lipid accumulation and increasing GSH and adenosine methionine levels by increasing homocysteine metabolism in mice and rats (Sim et al., 2015) (**Figure 2e**).

The participation of L-serine in the regulation of signaling pathways can not only relieve stress but also participate in the regulation of the inflammatory response. Studies have shown that L-serine decreases the production of IL-1β, TNF-α, IL-6 and IL-8 through the AMPK and nuclear factor kappa-B (NF-κB) signaling pathways, thereby reducing most of the inflammatory response in the host (Zhou et al., 2018b; Zhou et al., 2018c). Notably, we recently discovered that exogenous L-serine reduces macrophage and neutrophil-mediated lung inflammation in mice infected with *P. multocida* (He et al., 2019), and the underlying mechanism might be related to the macrophage inflammasome (unpublished data). Besides, this review highlights the great potential of L-serine in counteracting the growing threat of excessive inflammation (**Figures 2f, g**).

### 3.2 Significance of L-serine metabolism in the treatment of respiratory disease

#### 3.2.1 Infectious diseases

**Figure 3:**
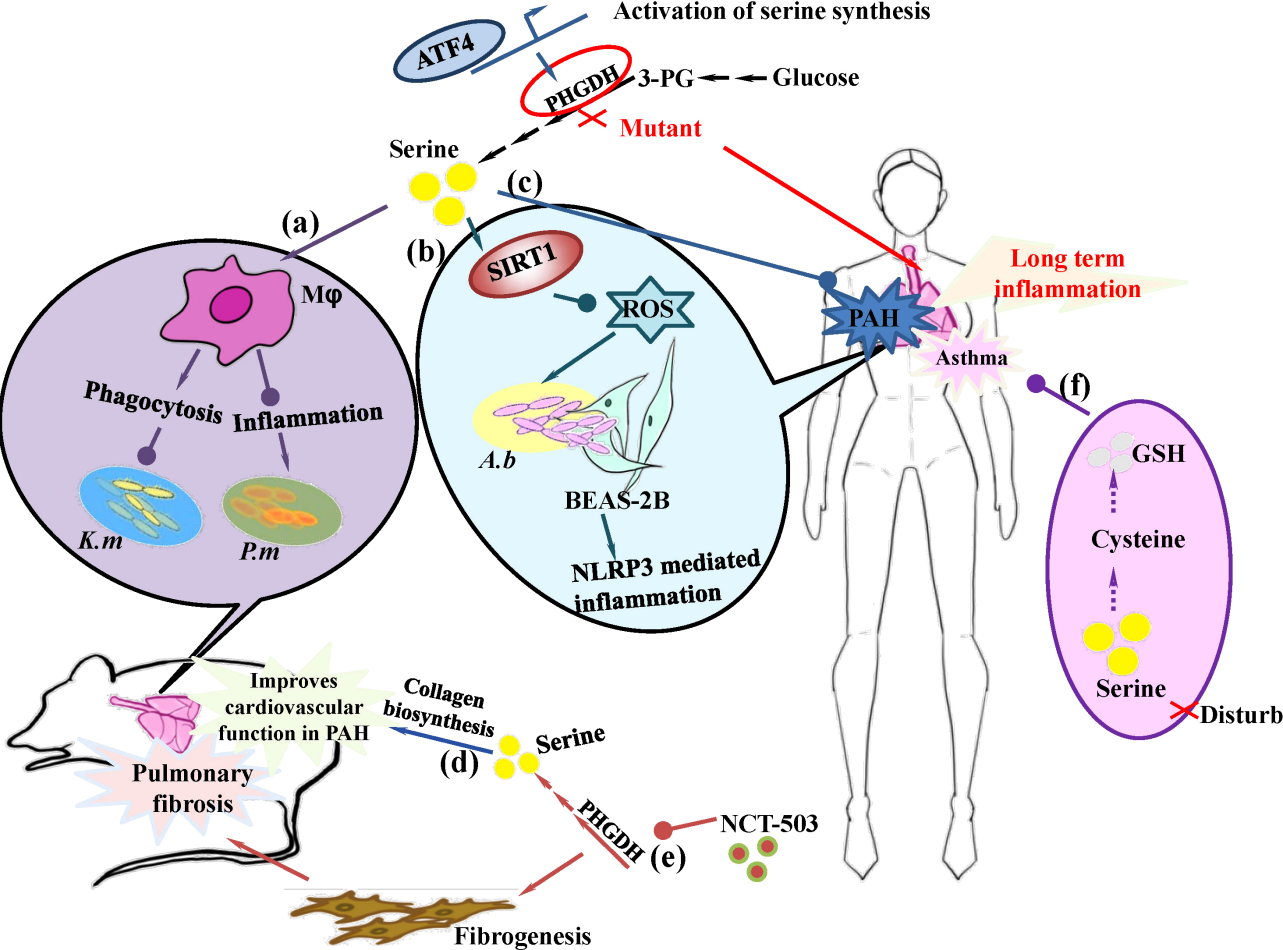
The role of L-serine and metabolism on the treatment of respiratory diseases. **(a)** L-serine enhances phagocytosis and decreases inflammation to help treat respiratory infectious diseases, such as, *Klebsiella pneumoniae* and *Pasteurella multocida*, in a mouse model. **(b)** L-serine upregulates SIRT1 to inhibit NLRP3 activation and decrease inflammation in *Acinetobacter baumannii* infected Beas 2B cells (human alveolar epithelial cells). **(c)** Serine and its metabolites serve as biomarkers of PAH. **(d)** Dairy intake of L-serine promotes collagen biosynthesis and improves cardiovascular fitness to treat cardiopulmonary vascular disease. **(e)** Inhibition of PHGDH impairs serine biosynthesis inhibits fibrogenesis and attenuates lung fibrosis in mice. **(f)** Disturbance of serine biosynthesis may alter cysteine and glutathione redox balance and contribute to asthma progression.

In addition to the regulation of inflammatory response, L-serine was found to be a potential protective substance to pulmonary infections. For example, metabolomics analysis of the liver metabolic profile of L-leucine treated tilapia during *Streptococcus iniae* infection reveals that serine is one of the two key metabolites. Exogenous L-serine reduces the mortality of tilapia infected with *S. iniae* (Du et al., 2017). Moreover, the intraperitoneal injection of L-serine in mice could reduce the load of *Klebsiella pneumoniae* in the infected lung and increase mouse survival, which might be attributed to the promotion of macrophage phagocytosis and provide a natural way to promote host clearance of lung pathogens (Liu et al., 2018) (**Figure 3a**). Our findings also revealed that exogenous L-serine supplementation significantly increased the survival rate of mice and decreased the colonization of *P. multocida* in the lungs, mainly through the alleviation of macrophage- and neutrophil-mediated inflammation in infected lungs (He et al., 2019) (**Figure 3a**). Moreover, researchers have revealed that, compared with those in healthy, COVID-19 positive asymptomatic pregnant females, serine metabolic pathways are upregulated and increase along with increasing severity (Hora et al., 2023). Consequently, D‐serine, phenylacetaldehyde, and pyruvate were upregulated in pregnant women with COVID‐19, which was also positively correlated with IL‐9 in the mild group. An Egyptian girl born to consanguineous parents was identified to have a homozygous mutation in PHGDH, resulting in recurrent episodes of prolonged and severe chest infections (Zaki et al., 2017). Similarly, Zhou et al. revealed that decreased L-serine levels were identified as the most crucial metabolic biomarker in low-virulent *Acinetobacter baumannii* strains compare to that of high-virulent strains. L-serine can reduce the virulence gene expression of *A. baumannii* in Beas 2B cells and inhibit the activation of the NLRP3 inflammasome by decreasing the generation of ROS and mtROS and lowering the release of inflammatory cytokines (IL-18 and IL-1β) through the upregulation of SIRT1 (Zhou et al., 2024) (**Figure 3b**). Taken together, these findings suggest that L-serine plays a role in the anti-infection of hosts and can serve as a novel strategy for the treatment of many pathogens. However, the underlying mechanisms still need to be elucidated.

#### 3.2.2 Pulmonary arterial hypertension

Pulmonary arterial hypertension (PAH) is a multifactorial, chronic disease process that results in pulmonary arterial endothelial dysfunction and smooth muscular hypertrophy, leading to right ventricular failure and even death (Humbert et al., 2023; Mocumbi et al., 2024; Provencher et al., 2024). Although many molecular pathways related to PAH, such as endothelin-1 dependent (Pulido et al., 2013), prostacyclin-mediated (Tuder et al., 1999), vascular calcium channels (Sitbon et al., 2005) and nitric oxide driven pathways (Tonelli et al., 2013) have been extensively studied and applied to alleviate patients’ pain, overall mortality in PAH patients has not significantly changed (Shah et al., 2022). The newly identified features of the disease increasingly regard PAH as a multipronged disease involving multiple points of interaction between genetics, metabolomics, imbalance of vasoconstrictor and vasodilator responses, endothelial and smooth muscle dysfunction, thrombosis and platelet dysregulation, and mitochondrial and miRNA abnormalities. Recently, research based on a Mendelian randomization study revealed that of 574 metabolites, serine was negatively associated with the clinical severity of PAH (Alhathli et al., 2024) (**Figure 3C**). Rare variant analysis has revealed that loss-of-function mutations within activating transcription factor 4 (ATF4), a transcription factor responsible for the upregulation of serine synthesis under conditions of serine starvation, are associated with higher risk for PAH, which further suggests serine is closely related PAH. Moreover, diary intake of serine can facilitate YAP- and TAZ-driven glutamine and serine catabolism to sustain proline and glycine anabolism and promote collagen biosynthesis, which improves cardiovascular function in PAH rodent models (Rachedi et al., 2024) (**Figure 3d**). These evidences suggest that amino acids, especially serine, are very important for the homeostasis of pulmonary arterial fitness.

#### 3.2.3 Pulmonary fibrosis

Pulmonary fibrosis is a chronic progressive disorder and the most common interstitial lung disease; it progresses with the accumulation of extracellular matrix (ECM) proteins such as collagen, along with the recruitment of fibroblasts and myofibroblasts (Rajesh et al., 2023). The activation of myofibroblasts involves further metabolic remodeling to support biosynthetic requirements, such as collagen. Transforming growth factor (TGF)-β, a key cytokine that promotes fibrogenesis, is upstream of PHGDH, and knockdown of SMAD3 can reduce TGF-β-induced PHGDH expression to impair collagen protein synthesis (Nigdelioglu et al., 2016). Robert et al. further revealed that inhibiting PHGDH via NCT-503 can inhibit fibrogenesis and attenuate lung fibrosis in mice (Hamanaka et al., 2018) (**Figure 3e**). Glycine is one of the major amino acids that forms collagen and is pivotal for collagen synthesis (Hamanaka and Mutlu, 2021). As L-serine is one of the major glycine synthesis pathways *in vivo*, thus, targeting amino acids, such as L-serine could be a potential approach for pulmonary fibrosis treatment (Miguel et al., 2024; Rajesh et al., 2023). Another recent study based on Raman spectroscopy and comparative machine learning suggested that metabolites with immune and inflammatory functions, such as serine, can serve as the top predictors of lung fibrosis and pneumonitis (Wiebe et al., 2024). Consistently, serum metabolic analysis of the anti-pulmonary fibrosis effects of Shuangshen Pingfei Formula (SSPF) on bleomycin-induced pulmonary fibrosis in rats revealed that serine may be a useful biomarker for pulmonary fibrosis treatment (Chen et al., 2022).

#### 3.2.4 Asthma

Asthma is a serious health and socioeconomic issue worldwide (Maciag and Phipatanakul, 2020; Masoli et al., 2004), and is no longer regarded as a single disease (Anderson, 2008). Researchers have focused on the identification of key metabolites useful for the diagnosis, monitoring and treatment of asthma (Maniscalco et al., 2019). A recent study on the metabolic features of exacerbating atopic asthma in children revealed that 103 metabolites, including serine, significantly differ from those in children with stable asthma. Consequently, the metabolite pathway, such as glycine, serine, and threonine metabolism is significantly enriched (Cottrill et al., 2023). Serine is a precursor of cysteine and is involved in the synthesis of glutathione. Disturbances in serine biosynthesis may account for alterations in the cysteine and glutathione redox balance, which are closely related to severe asthma in children (Fitzpatrick et al., 2011; Stephenson et al., 2015) (**Figure 3f**). Another study that combined whole blood transcriptome and serum metabolite analysis also demonstrated that serine and its metabolic pathway are negatively correlated with exposure to air pollutants such as PM2.5 and NO_2_ with childhood asthma history (Liao et al., 2022). During treatment of OVA-induced allergic asthma with traditional Chinese medicines, such as Dingchuan Decoction (DCD) (Li et al., 2024) and Nepeta bracteata (DBJJ, Dabao Jingjie in Chinese) (Abulaiti et al., 2024), the metabolite serine and its related pathways are both therapeutic targets.

#### 3.2.5 Lung related cancer

In addition to participating in the body’s metabolic network, the L-serine metabolic network is often hijacked by tumors to promote cancer cell proliferation. For example, cancer cells resynthesize serine through glucose or rely on foreign serine to promote cell proliferation (Possemato et al., 2011). Conversely, limiting L-serine supplementation can effectively inhibit the growth and proliferation of tumor cells (Maddocks et al., 2013).

As mentioned earlier, PHGDH is critical for L-serine biosynthesis, and its high expression in diseases, such as cancers, increase the throughput of serine synthesis (Possemato et al., 2011; Shen et al., 2022). In low-glucose treated bronchial epithelial cells, the conversion of serine to glycine is consistently increased, along with the upregulation of the mitochondrial one-carbon metabolism enzymes, serine hydroxymethyltransferase (SHMT2) and methylenetetrahydrofolate dehydrogenase (MTHFD2) (Haitzmann et al., 2024). Furthermore, the contribution of de nova synthesis of serine dramatically increased under low serine/glycine conditions. Consequently, inhibited PHGDH combined with suppressed pyruvate kinase (PK) M2 can inhibit cancer cell proliferation and induce G2/M phase arrest in non-small cell lung cancer A549 cells (Wang et al., 2023). During lung adenocarcinoma (LUAD) development, CBX4, a chromobox protein facilitates PHGDH transcription through interaction with GCN5, inducing increased histone acetylation on the PHGDH promoter, subsequently increasing serine biosynthesis and promoting LUAD proliferation (Zhao et al., 2024). Moreover, the overexpression of PHGDH in a mouse model resulted in resistance to erlotinib in xenografts, and the knockdown of PHGDH rescued the tumoricidal effect and restored sensitivity to erlotinib in both cell lines and xenografts (Dong et al., 2018). Interestingly, although increased PHGDH expression supports cancer cell proliferation, studies have demonstrated that low PHGDH induces abnormal protein glycosylation through activation of the hexosamine-sialic acid pathway, thus non-catalytically enhancing cancer spread and metastasis (Rossi et al., 2022). In summary, aberrant activation or dysregulation of L-serine-related metabolic enzymes and metabolic pathways is an important mechanism that promotes the malignant progression of tumors, and the heterogeneity of PHDGH in tumors may be a marker of tumor metastasis. For more information, a systematic review summarized the role of serine metabolism on the oncogenesis and treatment of lung cancers (Zhou et al., 2022b).

## 4 Concluding remarks and future perspective

L-serine has a variety of physiological functions, including the synthesis of amino acids, nucleotides, and lipids; DNA methylation; and protein phosphorylation. Thus, L-serine metabolism plays a vital role in the hemostasis of individuals, especially its function, which is closely related to the immune system, thus, L-serine is an important biomarker and treatment target. With the rapid development of metabolomics, this method enables us to capture the simultaneous status of many small-molecule metabolites and uncovers that serine is closely related to various diseases, including infectious diseases, lung fibrosis, pulmonary hypertension, asthma and lung cancers. Overall, this review highlights serine as a novel and feasible preventive and therapeutic option for tackling the increasing threat. However, most studies on serine for diseases treatment have been carried out in animals, and reproducibility and systematization in human trails are lacking. To draw more definitive conclusions, further research and verification should be carried out, such as controlling the sample type, dividing the disease course, expanding the sample size, and extending the follow-up period.
